# Shaping Oxidation Catalysis of Multivalent Mixed Oxides by Dedicated Hydrogen Pretreatment

**DOI:** 10.1021/acs.accounts.5c00428

**Published:** 2025-08-25

**Authors:** Herbert Over

**Affiliations:** † Institute of Physical Chemistry, 9175 Justus Liebig University, Heinrich-Buff-Ring 17, D-35392 Giessen, Germany; ‡ Center for Materials Research, Justus Liebig University, Heinrich-Buff-Ring 16, D-35392 Giessen, Germany

## Abstract

Supported metal nanoparticles used in heterogeneous catalysis can be prepared by using various methods, including deposition–precipitation and wet-chemical impregnation. The formed metal particles oxidize during the calcination step, which is required to burn off the organic components of the metal precursors. Therefore, the final step in metal catalyst preparation is always a high-temperature hydrogen treatment.

This Account discusses two rational hydrogen treatment methods capable of shaping the catalytic oxidation properties of a multivalent mixed oxide. The first example consists of mixed oxides with a perovskite structure ABO_3_, where a nobler metal replaces some of the B sites, such as Ru replacing Fe in LaFe_1–*x*_Ru_*x*_O_3_ (LFRO). High-temperature hydrogenation of this material at 800 °C results in the extraction of the more noble metal ion Ru^3+^, forming stable anchored Ru nanoparticles on the LFRO surface without affecting the structural integrity of the mixed oxide. This process is called exsolution and allows for precise control of metal particle size distribution. However, this process has two limitations: The exsolved Ru particles are passivated by an ultrathin LaO_*x*_ layer, and most of the Ru remains in the bulk of the host perovskite oxide and does not contribute to the catalytic activity. Based on a detailed microscopic knowledge, a dedicated redox protocol is developed that produces a catalyst in which most of LFRO’s Ru can be extracted by exsolution. This protocol ensures that the high concentration of small Ru particles is not passivated by LaO_*x*_ layers. The resulting catalyst exhibits superior catalytic activity in propane combustion and in CO_2_ reduction; in the latter, the selectivity shifts from CO to methane.

Second, I present a novel and versatile strategy to promote catalytic oxidation reactions by incorporating hydrogen into mixed oxides. The mixed oxide is designed to consist of one metal oxide (RuO_2_ or IrO_2_) that can activate the H_2_ dissociation process and a second component (rutile TiO_2_) that stabilizes the mixed oxide against in-depth chemical reduction when exposed to H_2_ at temperatures ranging from 150 to 250 °C. The resulting synergistic effect enables the mixed oxide to accumulate high concentrations of 20–30 atom % of incorporated H in its bulk while maintaining structural integrity. The incorporation of hydrogen has been shown to induce (macro, micro) strain within the mixed oxide lattice and modulate the electronic structure. These phenomena boost the oxidation activity in both thermo- and electrocatalysis, as demonstrated by catalytic propane combustion and the oxygen evolution reaction under acidic conditions.

## Graphical abstract

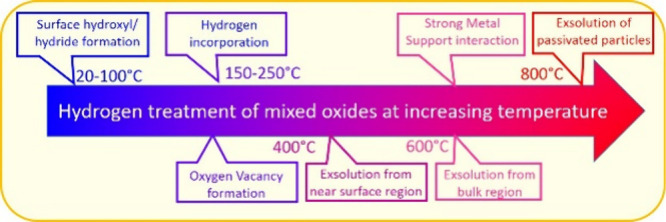

## Key References

-

Wang, W. ; Timmer, P. ; Spriewald Luciano, A. ; Wang, Y. ; Weber, T. ; Glatthaar, L. ; Guo, Y. ; Smarsly, B. M. ; Over, H. Inserted Hydrogen Promotes Oxidation Catalysis of Mixed Ru_0.3_Ti_0.7_O_2_ as Exemplified with Total Propane Oxidation and the HCl Oxidation Reaction. Catal. Sci. Technol. 2023, 13, 1395–1408 .^1^ Introduces the concept of hydrogen promotion for mixed oxide Ru_30 (Ru_*x*_Ti_1–*x*_O_2_) which is exemplified with catalytic propane combustion.
-

Wang, W. ; Zlatar, M. ; Wang, Y. ; Timmer, P. ; Spriewald Luciano, A. ; Glatthaar, L. ; Cherevko, S. ; Over, H. Hydrogenation of Mixed Ir-Ti Oxide, a Powerful Concept to Promote the Oxygen Evolution Reaction in Acidic Water Electrolysis. ACS Catal. 2025, 15, 6721–6730 .^2^ Shows for phase-pure 30 at% Ir in rutile TiO_2_ (Ir_30_pp) how hydrogenation improves thermo- and electro-oxidation catalysis, focusing on the electrocatalytic oxygen evolution reaction for acidic water electrolysis.
-

Wang, Y. ; Paciok, P. ; Pielsticker, L. ; Wang, W. ; Spriewald Luciano, A. ; Ding, M. ; Glatthaar, L. ; Hetaba, W. ; Guo, Y. ; Gallego, J. ; Smarsly, B. M. ; Over, H. Microscopic Insight into Ruthenium Exsolution from LaFe_0.9_Ru_0.1_O_3_ Perovskite. Chem. Mater. 2024, 36, 6246–6256 .^3^ Uncovers microscopic processes in the exsolution of Ru particles from LaFe_1–*x*_Ru_*x*_O_3_ (LFRO) including the formation and the removal of a passivating LaO_*x*_ layer.
-

Wang, Y. ; Paciok, P. ; Pielsticker, L. ; Spriewald Luciano, A. ; Glatthaar, L. ; Xu, A. ; He, Z. ; Ding, M. ; Hetaba, Y. ; Gallego, W. ; Guo, J. ; Smarsly, B. M. ; Over, H. Boosting Ru Atomic Efficiency of LaFe_0.97_Ru_0.03_O_3_ via Knowledge-Driven Synthesis Design. Chem. Sci. 2025, 16, 7739–7750 40201168 10.1039/d5sc00778jPMC11973924.^4^ Based on fundamental knowledge, a high-performing Ru catalyst is prepared by a high temperature redox treatment followed by mild reduction to exsolve most of the active component Ru into a distribution of small, stable particles.

## 1 Introduction

In catalysis research, optimizing catalyst materials for a specific reactions is key in terms of activity, selectivity, and stability.^5,6^ For precious metal catalysts, the surface-to-volume ratio of the metal atoms (i.e., the dispersion) should be as high as possible, ensuring that as many expensive metal atoms as possible are on the surface, ready to contribute to the catalytic reaction. To stabilize a high dispersion of metal particles, the active metal particles are supported by a carrier material. Catalyst activity roughly scales with the number of surface metal atoms and, consequently, with dispersion. To maintain high activity and stability, the active particles must be stabilized against sintering^7^ and chemical transformation,^8^ while the intrinsic activity of metal particles can be fine-tuned by promoters that do not directly participate in the catalytic reaction.^9^

Oxides are often used for oxidation catalysis and are typically employed as carriers that stabilize highly dispersed active metal particles. However, oxides can also serve directly as active components. The functionality of oxides can be increased by using mixed oxides—more precisely, a solid solution of two or more oxides, where one component comprises the catalytically active component.^10^ Examples include Ru_*x*_Ti_1–*x*_O_2_, Ir_*x*_Ti_1–*x*_O_2_, and LaFe_1–*x*_Ru_*x*_O_3_ (LFR*x*), which are discussed in this Account. Mixed oxides have several potential benefits over supported catalysts on carrier oxides, including improved stability, synergy effects, higher selectivity, and electronic conductivity. However, there is a trade-off: lower mass activity. Most of the active component is buried in the bulk of the mixed oxide, so it does not contribute to the catalytic conversion. Mass activity increases when mixed oxide particles are small. Additionally, the mass activity can be increased by enriching the near-surface concentration of the mixed oxide particle with the active component. In an extreme case, the active component can be stabilized as a single atom on the oxide surface, which maximizes dispersion.^11–13^

In this Account, I will focus on oxidation catalysis and explain how pretreating the mixed oxide catalyst with hydrogen at different temperatures improves its catalytic oxidation performance. I will discuss two seemingly unrelated examples that improve the performance of catalytic oxidation when pretreated with hydrogen, resulting in metastable catalysts for oxidation reactions. First, I will consider mixed perovskite oxides ABO_3_, where the B site is partially substituted with ruthenium, the active component. Hydrogenation at temperatures up to 800 °C results in the formation of socketed Ru particles with a narrow size distribution that remain stable during high-temperature reduction reactions and medium-temperature propane oxidation reactions. Second, mild hydrogenation of a solid solution of RuO_2_ or IrO_2_ with rutile TiO_2_ (r-TiO_2_) at 150–250 °C can incorporate hydrogen into the mixed oxide. This promotes the activity of various catalytic oxidation reactions, including propane oxidation and the oxygen evolution reaction (OER) in acidic water electrolysis.

## 2 General Aspects of Hydrogen Interaction with (Mixed) Oxide and Metal Samples

Here I discuss the interaction of hydrogen with mixed oxides and metals at various temperatures from a general point of view. I would like to encourage readers to take a closer look at hydrogen treatments in catalysts, as the effect on mixed oxides varies greatly depending on the temperature. As indicated by the H_2_ molecular orbital (MO) diagram in Figure 1, the transfer of an additional electron from the proper metal state to the antibonding state (σ*) of H_2_ readily leads to its dissociation. Upon dissociation, the interaction between the H atoms and the solid surface becomes quite strong, reaching a strength of chemisorption that is substantially higher than half the binding energy of H_2_. H_2_ does not serve as a strong reducing agent because an adsorbed hydrogen atom can donate or accept an electron.

**Figure 1:**
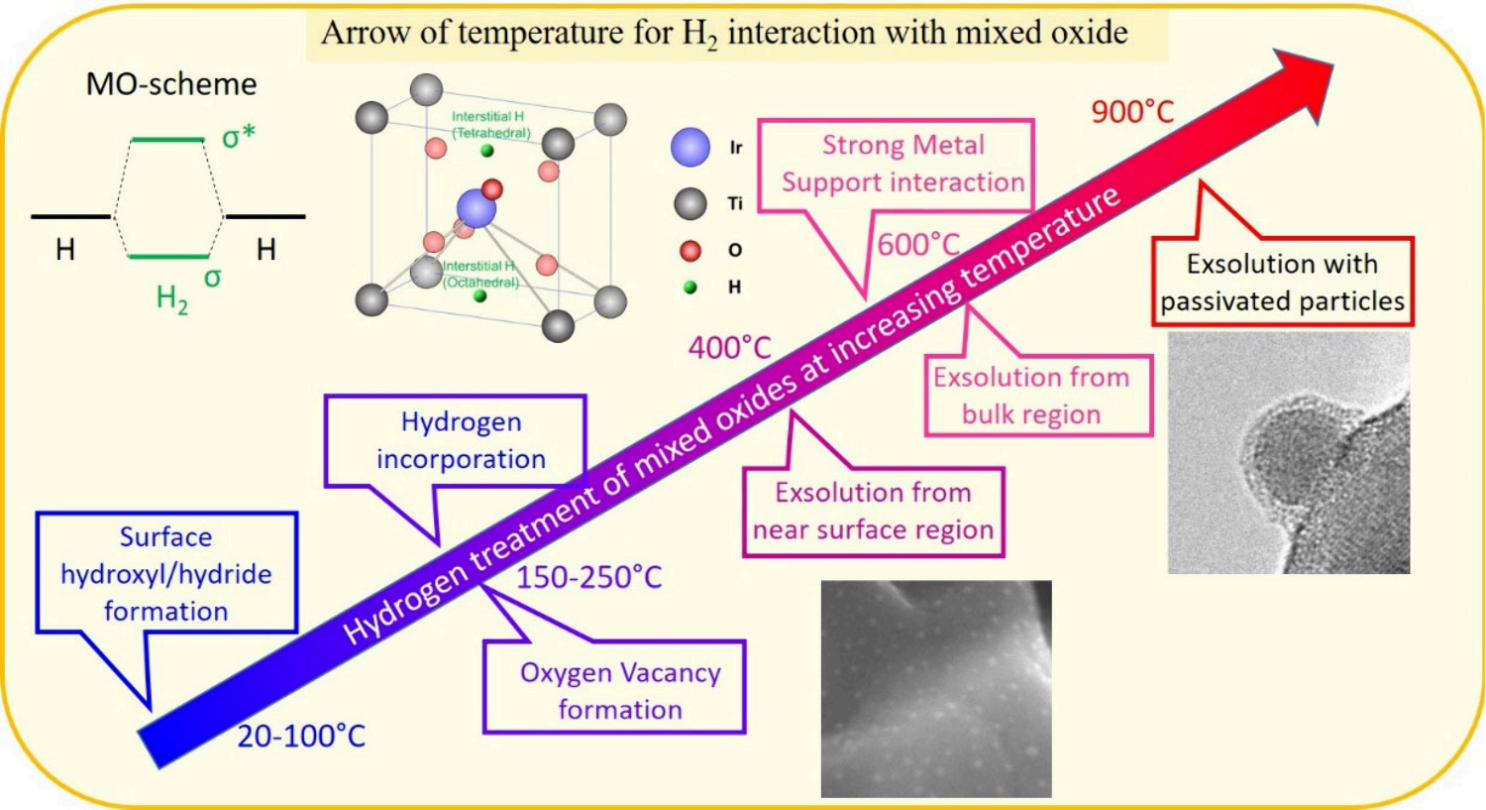
MO scheme of H_2_. Processes of mixed oxides with multivalent metal ions occurring during hydrogen exposure at various temperatures.

Many metals, particularly transition metals, can activate H_2_ dissociation.^14^ Upon dissociation of H_2_, the work function often increases, indicating that adsorbed H on metal surfaces is in the −δ state (a kind of hydride species). This is consistent with hydrogen’s higher Pauling electronegativity of 2.2 than that of most transition metals, which have an electronegativity lower than 2.

Depending on the chemical nature of the oxide, oxide surfaces can split H_2_ by either heterolytic or homolytic dissociation.^15^ Heterolytic cleavage of the H–H bond forms surface hydroxyl (O^δ−^–H^δ+^) and surface hydride (M^δ+^–H^δ−^) species, leaving the oxidation state of the metal ion unaffected. Homolytic dissociation, on the other hand, produces two OH surface groups and reduces two metal centers, either isolated or delocalized in the conduction band of the material, to establish charge compensation. At low temperatures of H_2_ exposure, these are the only species observed to form on the oxide surfaces. For RuO_2_(110), heterolytic dissociation of H_2_ is observed at low temperatures (150 K), finally transforming to only O–H species at room temperature.^16^ H_2_ molecules are much more difficult to activate on the rutile-TiO_2_ (r-TiO_2_) surface than on RuO_2_ or IrO_2_,^17^ so that an active component is needed to dissociate H_2_, and via back spillover, hydrogen can cover the r-TiO_2_ surface.^18^

Due to their small size, H atoms can readily move into the interior of various metals. Therefore, H atoms can form stable hydride compounds with most metals, which can be ionic, covalent, or metallic depending on the metal’s position in the periodic table.^14^ Hydrogen incorporation has been reported less frequently for oxides. For example, in r-TiO_2_, hydrogen can form deep donor states through O–H^+^ complexes or H species in O vacancies,^19,20^ although the concentrations are very low. Hydrogen incorporation into RuO_2_ and IrO_2_ from H_2_ exposure is unknown. Instead, RuO_2_ ^21,22^ and IrO_2_ ^2^ fully reduce to the metal state when exposed to H_2_ at temperatures around 200–300 °C. In contrast, r-TiO_2_ is not reduced under these mild conditions. Much higher reduction temperatures of 600–1000 °C are needed to form reduced TiO_2_ phases, including Magnéli phases down to Ti_2_O_3_.^23,24^

Figure 1 summarizes the processes that occur when mixed oxides are exposed to H_2_, while retaining their structural integrity. Depending on the applied reduction temperature, a continuous switch in relevant processes is observed: from adsorption (surface hydroxyl and hydride formation),^15^ to incorporation,^25^ to oxygen vacancy formation,^3,26,27^ and finally, to the reduction of one component of the mixed oxide and the exsolution of particles,^28–30^ The exsolved particles may be covered by a passivating oxide layer^31,32^ due to strong metal support interactions: SMSIs.^33–35^ The flexibility and versatility of H_2_ treatment of mixed oxides is remarkable. Under pure O_2_ conditions, these H_2_-induced processes are largely reversible, depending on the applied temperature. However, under oxidizing reaction conditions, hydrogen-induced modifications, such as incorporation and exsolution, can be *metastable*.^1,31^ Most of these hydrogen-induced processes are operative in the Ru exsolution process of LaFe_1–*x*_Ru_*x*_O_3_.

## 3 Ruthenium Exsolution from LaFe_1–*x*_Ru_*x*_O_3_ Perovskite by High-Temperature H_2_ Treatment

LaFeO_3_ (LFO) is a simple perovskite, (ABO_3_; cf. Figure 2a) consisting of only three elements. It can accommodate a variety of defects and can partially substitute the B sites with the target metal ion to be exsolved while retaining structural integrity.^36^ However, preparing LFO requires temperatures of 900 °C, resulting in a final specific surface area of only about 10 m^2^/g. The iron (Fe) in the B site of the perovskite LaFeO_3_ can be partially replaced by ruthenium (Ru), which is a nobler metal than iron. The maximum amount of incorporated ruthenium (Ru) is about 30 atomic percent (atom %) before phase separation of ruthenium dioxide (RuO_2_) as a second phase occurs.^32^ Ten atomic percent (10 atom %) of ruthenium replacement (LFR10) introduces local strain that leads to broadening and shifting of vibration features in the Raman spectra.^3,32^ However, the X-ray diffraction pattern is identical to that of the LFO (Figure 2b).

**Figure 2:**
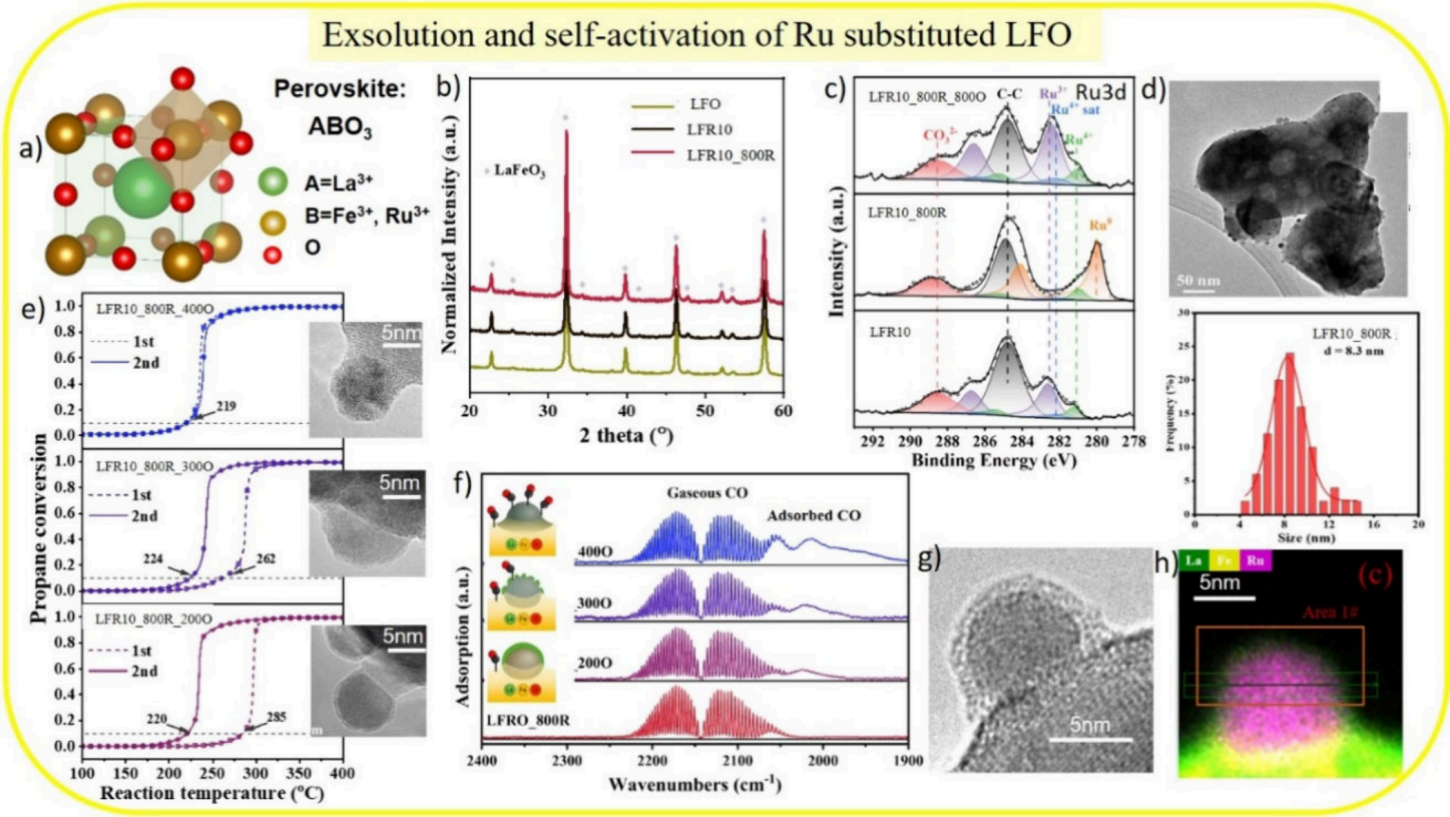
(a) Structure model of the ABO_3_ perovskite: LaFe_0.9_Ru_0.1_O_3_ (LFR10), 10 atom % of Fe on the B-sites is substituted by Ru. (b) Powder X-ray diffraction pattern of LFR10 before and after H_2_ treatment (4 vol % H_2_/Ar, 100 mL/min) at 800 °C for 3 h (LFR10_800R) compared to LaFeO_3_ (LFO). (c) Ru 3d XP-spectra of LFR10 and LFR10_800R before and after oxidation at 800 °C (LFR10_800R_800O). (d) TEM micrograph with size distribution. (e) Two consecutive light-off curves (1st and 2nd) for propane combustion (1 vol % C_3_H_8_, 10 vol % O_2_, and 89 vol % N_2_; 100 mL/min) together with high-resolution TEM images. (f) CO DRIFT-spectra of the exsolved Ru particle for LFR10_800R mildly reoxidized at 200 °C (LFR10_800R_200O), 300 °C (_300O), and 400 °C (_400O) with 10%O_2_/Ar. (g) High-resolution TEM micrograph and (h) element mapping of an exsolved Ru particle of LFR10_800R. Adapted with permission from ref 32. Copyright 2023 Elsevier.

The composition of the near-surface region can be quantified by using X-ray photoelectron spectroscopy (XPS) of the Ru 3d core level (Figure 2c). Ruthenium is mostly in the +3 state, as expected when Ru substitutes Fe^3+^. A small amount of ruthenium is in the β-state, which has a lower oxidation state than +3. Upon hydrogenation at 800 °C, the Ru^3+^ signal disappears in the Ru 3d spectrum. Instead, a new Ru^0^ feature with an oxidation state of zero appears, revealing the exsolution of metallic Ru particles with an average size of 8 nm on the LFR10 surface, as corroborated by TEM experiments (Figure 2d). The X-ray diffraction pattern (Figure 2b) is identical to that of LFO, evidencing that the perovskite structure of LFR10 remains stable upon high-temperature hydrogenation. Upon reoxidation at 800 °C, LFR10_800R transforms back toward LFR10, which has no metallic Ru component and a dominant Ru^3+^ spectral feature in Ru 3d XPS (Figure 2c). This process of self-regeneration was originally introduced by Nishihata et al.^28^ Note that the high-temperature redox treatment enriches the surface of LFR10_800R_800O with ruthenium, increasing its concentration from 8 atom % (LFR10) to 13 atom %.^32^

The activity of LFR10_800R after exsolution was tested by using catalytic propane combustion (Figure 2d). Surprisingly, its activity during the initial light-off curve is low, similar to that of the LFR10_800R_200O after reoxidation at 200 °C. However, a second run of the conversion curve shows substantially higher activity, or *self-activation*, thereby reducing the T10 value (the temperature at which 10% conversion is reached) from 290 to 220 °C. The hysteresis of the conversion curves in Figure 2d gradually disappears upon reoxidation of LFR10_800R at temperatures up to 400 °C, and finally transient deactivation is suppressed with a 400O treatment. Note that the exsolved particles in the TEM micrographs are socketed. The T10 value for all samples in the second run of the conversion curve is 220 °C. High-resolution TEM (insets in Figure 2e,g) reveals that the deactivation is due to a capping layer covering the Ru particle. Mild reoxidation at 400 °C fully removes the capping layer. Therefore, the hysteresis in the conversion curves can be uniquely traced to the removal of the capping layer; or in other words, the hysteresis can diagnose the formation of a capping layer.

With TEM, only a limited area of the sample can be examined. To demonstrate that the passivating layer is a universal characteristic of all particles, a highly surface-sensitive averaging method must be employed: CO-DRIFTS (diffuse reflectance infrared Fourier transform spectroscopy).^37^ The adsorption of CO depends on the concentration of vacant Ru surface sites on the particle and on LFR10. The DRIFT spectrum of the LFR10_800R sample (Figure 2f) reveals only small spectral features around 2060 cm^–1^, which can be assigned to the CO stretch vibration of adsorbed CO on LFR10. As the reoxidation temperature increases, the bands of adsorbed CO increase. At 400 °C, the CO bands are fully developed, indicating that the capping layer has been removed. Element mapping at the nanoscale (Figure 2h) reveals that the capping layer is a La-containing compound, likely LaO_*x*_.

The remaining question is at what temperature the capping layer forms during the exsolution process. According to in situ TEM, a reduction temperature of 500 °C is sufficient to exsolve small Ru particles (Figure 3a,b). Ambient pressure XPS reveals metallic Ru^0^ after 500R, indicating exsolved Ru particles. Repetitive conversion curves during propane combustion do not indicate *self-activation*; in the first run, LFR10_500R is even slightly more active than that in subsequent runs. This means that no capping layer formed. CO-DRIFTS shows that, at 600 °C, the particles are partly covered by a capping layer but not at 550 °C. This finding is supported by repetitive conversion curves, where the second curve of LFR10_600R and LFR10_700R indicates a more active catalyst than the first run (hysteresis in Figure 3f,g).

**Figure 3:**
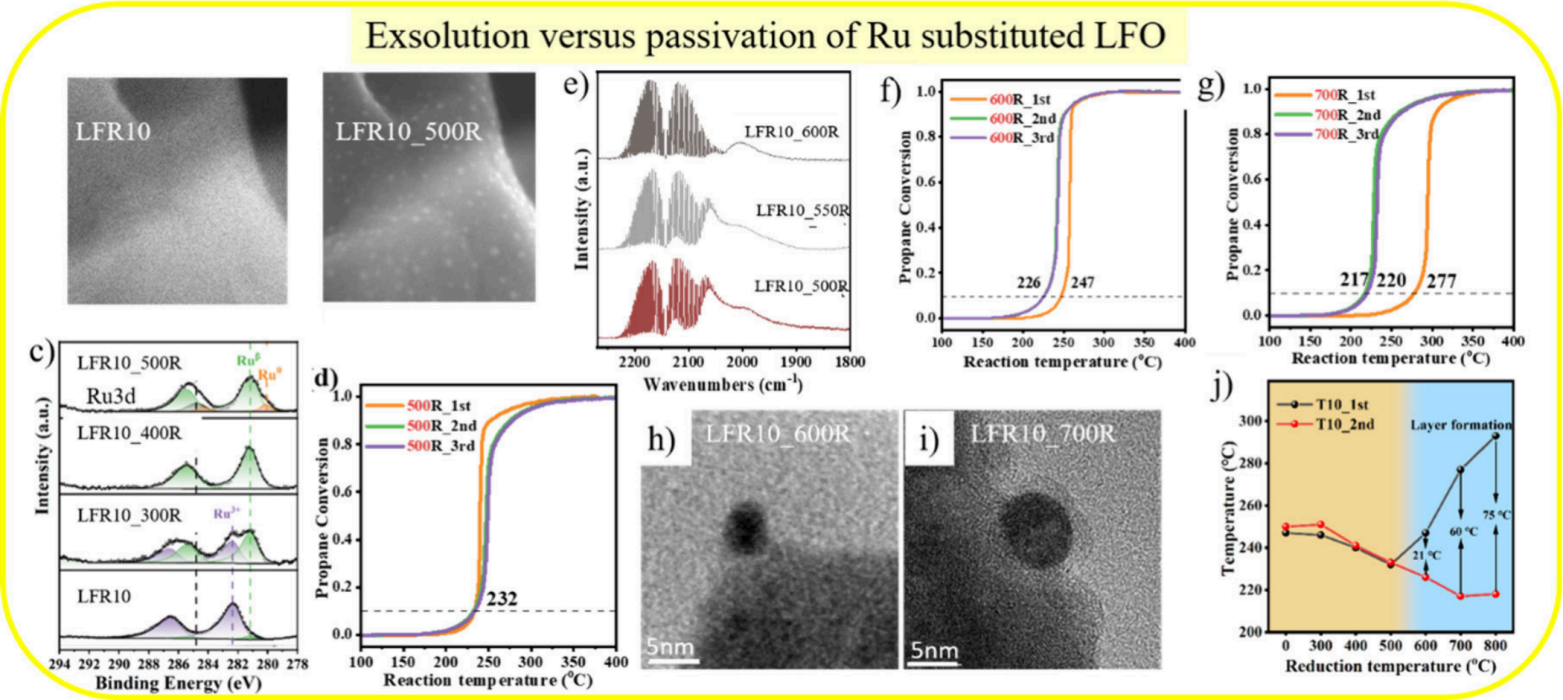
*In situ* secondary electron (SE)­TEM micrographs of LFR10 (a) at room temperature and (b) at 500 °C after H_2_ exposure for 20 min (LFR10_500R). (c) *In situ* ambient pressure Ru 3d XP-spectra of LFR10 and LFR10 reduced at various temperatures (300 °C, 400 °C, 500 °C) with 1 mbar H_2_. (d) Light-off curves of LFR10_500R (three consecutive cycles) for catalytic propane combustion (1 vol % C_3_H_8_, 10 vol % O_2_, and 89 vol % N_2_; 100 mL/min). (e) CO-DRIFT spectra of LFR10 after H_2_ treatment at 500 °C, 550 °C, 600 °C. Light-off curve of (f) LFR10_600R and (g) LFR10_700R for catalytic propane combustion. High-resolution TEM micrographs of (h) LFR10_600R and (i) LFR10_700R. (j) T10 value of two consecutive propane combustion tests of LFR10 that is reduced by H_2_ at various temperatures ranging from 300 to 800 °C. Adapted with permission from ref 3. Copyright 2024 American Chemical Society.

High-resolution TEM of LFR10_600R (Figure 3h,i) does not indicate capping-layer formation. However, at 700 °C, the capping layer is clearly discernible. The hystereses of conversion data are summarized in Figure 3j with the T10 values of the first and second runs. These experiments demonstrate that the capping layer forms only under hydrogenation above 600 °C. Overall, the exsolution process and the capping layer formation commence below 500 °C and above 600 °C, respectively.

For the next set of experiments, the Ru concentration at Fe sites is reduced from 10 atom % to 3 atom % (LaFe_0.97_Ru_0.03_O_2_: LFR3). This decrease is necessary to emphasize the effect of Ru enrichment in the near-surface region through a high-temperature redox treatment at 800 °C, as evidenced by the Ru 3d spectra in Figure 2c.

High-temperature redox treatment increases the Ru concentration in the near-surface region of LFR3_redox from 2.4 atom % (LFR3) to 6.8 atom % (Figure 4a,b). A final mild reduction treatment at 500 °C then induces Ru exsolution. As evident from TEM, the concentration of Ru particles in LFR3_Redox_500R is much higher than that in LFR3_500R (Figure 4c,d). Quantitative analysis reveals an average particle size of 2.3 nm in LFR3_500R and 1.9 nm in LFR3_Redox_500R. The Ru particle concentration increases from 4350 μm^–2^ to 26,000 μm^–2^, respectively. CO-DRIFTS indicates that no passivating layer forms at 500 °C (Figure 4e). Subsequently, the resulting catalysts are tested in propane combustion. LFR3_Redox_500R is substantially more active than LFR3_500R (Figure 4f).

**Figure 4:**
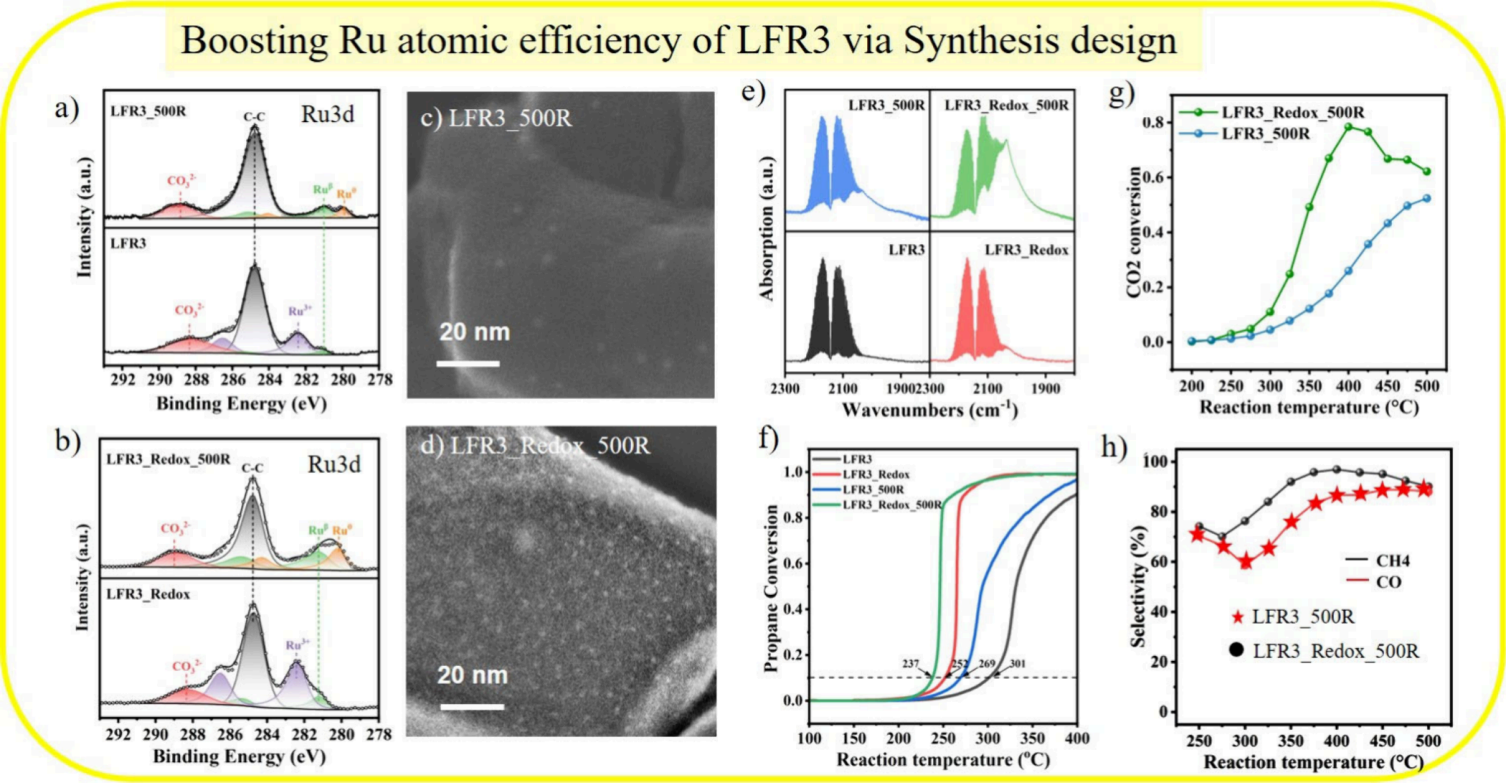
Fitted C 1s + Ru 3d *ex situ* XPS spectra of (a) LFR3, LFR3_500R, and (b) high-temperature redox treated samples LFR3_Redox and LFR3_Redox_500R together with TEM micrographs (c, d). (e) CO-DRIFT spectra quantify the concentration of active sites. (f) Conversion curves for catalytic propane combustion (1 vol % C_3_H_8_, 10 vol % O_2_, and 89 vol % N_2_; 100 mL/min) for LFR3 and various treatments of LFR3. Catalytic CO_2_ hydrogenation (4 sccm of CO_2_ and 16 sccm of H_2_, balanced by 20 sccm of Ar) over LFR3_500R compared to LFR3_Redox_500R as a function of temperature: (g) conversion and (h) selectivity. Reproduced from ref 4. Available under a CC BY 3.0 license. Copyright 2025 Royal Society of Chemistry.

The increase in LFR3_Redox_500R’s activity is even more pronounced in the CO_2_ hydrogenation reaction (Figure 4g). The selectivity switches from CO to CH_4_ when comparing LFR3_500R and LFR3_Redox_500R. This change in selectivity is explained by the higher rate of H_2_ activation and, consequently, the higher Ru particle concentration.^38,39^ However, no passivating LaO_*x*_ layer is observed to form at a reaction temperature of 500 °C.^4^

## 4 Hydrogen Incorporation into Phase-Pure Mixed Rutile Oxides by Low-Temperature H_2_ Treatment

A different hydrogenation treatment is pursued here with mixed rutile oxides of RuO_2_ or IrO_2_ and r-TiO_2_: the mixed oxide catalyst is hydrogenated at temperatures of 250 and 150 °C, respectively. It has been discovered that the hydrogenated catalyst is much more effective as oxidation catalyst than the initial mixed oxide catalyst.^1,2^ At first, this strategy of hydrogen incorporation seems counterintuitive since one would expect hydrogen-induced changes in the mixed oxide catalyst to be fully restored under oxidizing reaction conditions. However, this does not happen when the reaction conditions are properly chosen. There is a temperature range in which the incorporated hydrogen becomes metastable even under strongly oxidizing reaction conditions.

The incorporation of hydrogen into Ru_*x*_Ti_1–*x*_O depends on the concentration of the active component. The highest H loading is achieved with 60 atom % Ru.^25^ Similar results are expected for the Ir_*x*_Ti_1–*x*_O_2_ system, but they have not yet been studied.

In general, mixed rutile oxides of Ru_*x*_Ti_1–*x*_O_2_ ^1^ and Ir_*x*_Ti_1–*x*_O_2_ ^2^ have a large miscibility gap with phase separation into a solid solution and almost pure IrO_2_ or RuO_2_. To achieve phase-pure materials, one must remove the second phase either by leaching^40^ or by using a more sophisticated temperature protocol in the Pechini method.^41^ Here, we focus on phase-pure mixed oxides of 30 atom % IrO_2_ and RuO_2_ with r-TiO_2_. (Ru_30_pp and Ir_30_pp) This is the typical concentration of the active component in dimensionally stable anodes that maintain high electronic conductivity.^42^

### 4.1 Ru_0.3_Ti_0.7_O_2_ (Ru_30_pp)

Exposing the Ru_30_pp to H_2_ at 250 °C (Ru_30_pp_250R) results in hydrogen being incorporated into the mixed oxide lattice, as demonstrated by the shift of rutile XRD peaks. (Figure 5a) The total amount of incorporated hydrogen is 20 atom % as determined by TG-MS (Figure 5c). XPS reveals that the oxidation state of Ru is unaffected by this hydrogenation step (Figure 5b), as are the oxidation states of Ti and O.^40^ The only change in the Ru 3d spectra is the shift of the satellite peak to lower binding energies, which is indicative of a reduced electron density of the conduction band in the near surface region.^43^ The incorporated hydrogen is a labile species that can be removed by exposing the sample to O_2_ at 100 °C,^1^ which is consistent with the TG-MS experiment shown in Figure 5c.

**Figure 5:**
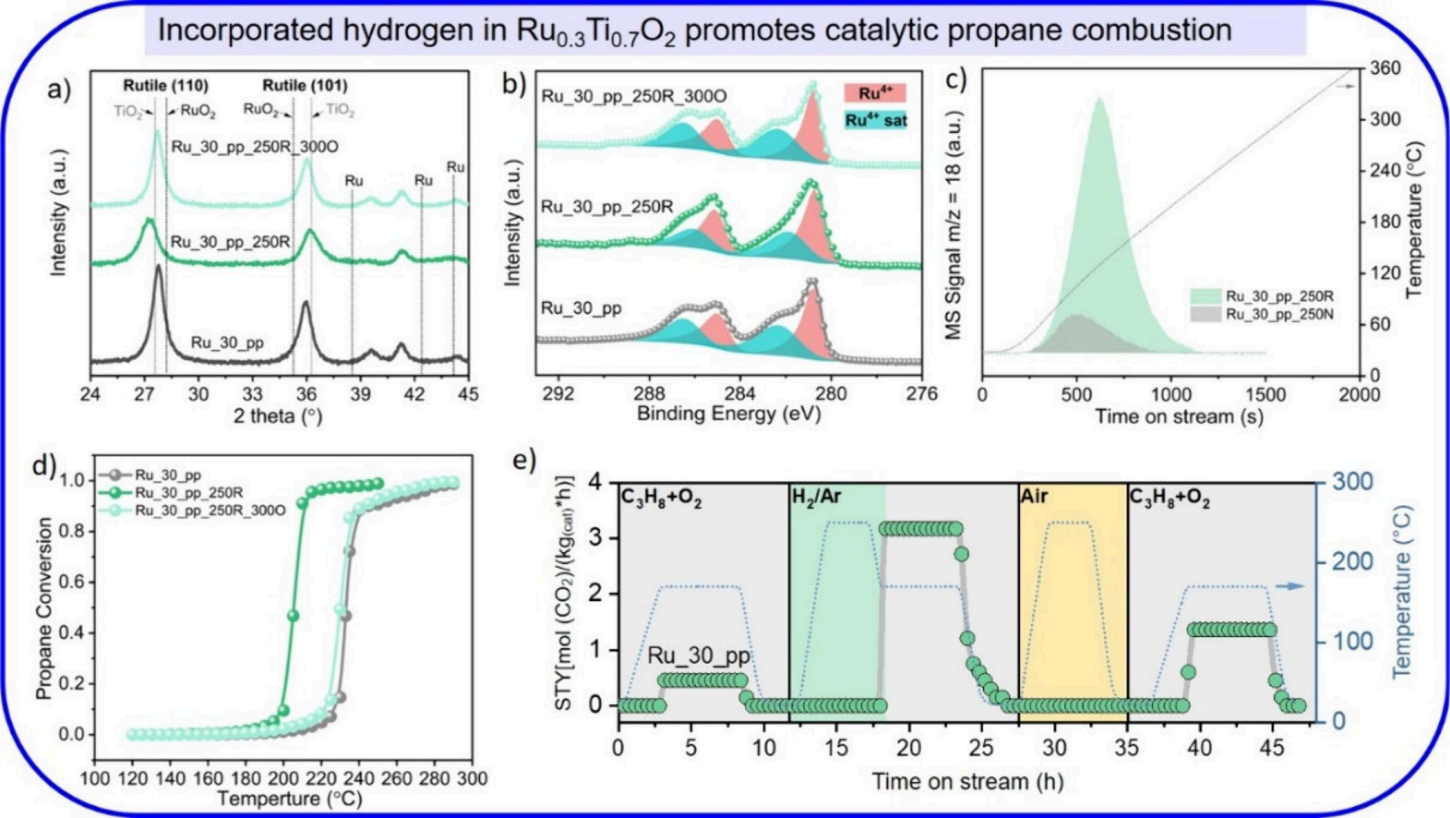
(a) Powder XRD pattern of phase-pure Ru_30_pp before and after reduction at 250 °C for 3 h in 4% H_2_ (Ru_30_pp_250R) compared to that after reoxidation at 300 °C (Ru_30_pp_250R_300O). (b) Ru 3d XP spectra of phase-pure Ru_30_pp before and after mild reduction at 150 °C for 3 h in 4% H_2_ (Ru_30_pp_250R) compared to that after reoxidation at 300 °C (Ru_30_pp_250R_300O). (c) Quantification of the mole fraction of inserted hydrogen into Ru_30_pp_150R by TG-MS; reference Ru_30_pp_150N (Ru_30_pp treated in N_2_ at 150 °C). (d) Catalytic oxidation activity of phase-pure Ru_30_pp, Ru_30_pp_250R, and Ru_30_pp_250R_300O in the combustion of propane. Activity data (STY: space time yield = mol product per hour and kg catalyst) of Ru_30_pp in the catalytic propane combustion (175 °C, 1 vol % C_3_H_8_, 5 vol % O_2_, balanced by N_2_; 100 sccm/min; gray) before and after the catalyst is in situ treated with hydrogen (4 vol % H_2_ in 96 vol % Ar: H_2_/Ar; green) or in air at 250 °C (yellow). Reproduced from ref 40. Available under a CC BY-NC-ND 4.0 license. Copyright 2025 Justus-Liebig-University Giessen.

Catalytic tests of propane combustion (Figure 5d) indicate that the 250R treatment substantially increases the activity, shifting the conversion curves 20 °C lower. Mild reoxidation at 300 °C restores the original structure, electronic properties, and catalytic propane combustion activity of Ru_0.3_Ti_0.7_O_2_ (Figure 5a,b,d). H_2_ exposure can also be conducted in situ (Figure 5e) by switching the reaction mixture in the flow reactor from propane combustion to pure H_2_. The activity substantially increases after in situ hydrogenation, while reoxidation at 250 °C leads to an activity decline, which clearly shows that the increase in activity is due to incorporated hydrogen.

### 4.2 Ir_0.3_Ti_0.7_O_2_ (Ir_30_pp)

Exposing the Ir_30_pp to H_2_ at 150 °C (Ir_30_pp_150R) results in hydrogen being incorporated into the mixed oxide lattice. This is evident from the slight shift of the rutile diffraction peaks (Figure 6a). The total amount of incorporated hydrogen is 30 atom % as quantified by TG-MS (Figure 6c). According to XPS, neither the oxidation state of iridium (Figure 5b) nor that of Ti and O is affected by this hydrogenation step.^2^ However, the satellite peak in Ir 4f disappears upon hydrogenation. The incorporated H is a labile species that disappears when the sample temperature is increased to 100 °C in air.^40^ Catalytic tests with the propane combustion (Figure 5d) indicate that the activity significantly increases with the 150R treatment,^2^ shifting the conversion curves by about 10 °C to lower temperatures. Mild reoxidation at 300 °C (Ir_30_pp_150R_300O) restores the structure, the propane oxidation activity, and the satellite peak of Ir_30_pp (Figure 6a,b,d).

**Figure 6:**
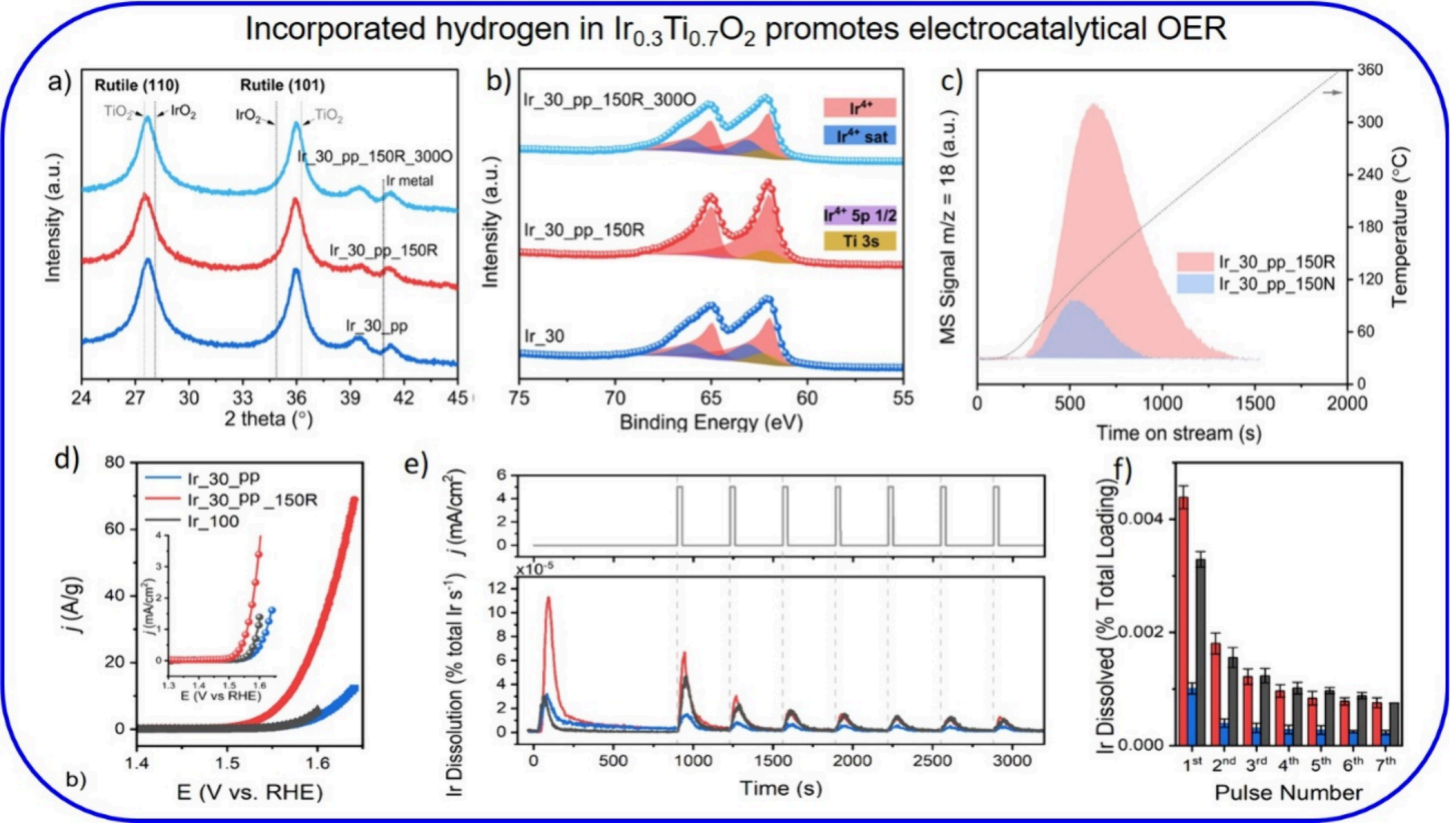
(a) Powder XRD-pattern and (b) Ir 4f XP spectra of phase-pure Ir_30_pp before and after mild reduction at 150 °C for 3 h in 4% H_2_ (Ir_30_pp_150R) compared with that after reoxidation at 300 °C (Ir_30_pp_150R_300O). (c) Quantification of the mole fraction of inserted hydrogen into Ir_30_pp_150R by TG-MS; reference Ir_30_pp_150N (Ir_30_pp treated in N_2_ at 150 °C). (d) OER activity of phase-pure Ir_30_pp and Ir_30_pp_150R (in comparison with Ir_100). (e) Ir dissolution of Ir_30_pp and Ir_30_pp_150R monitored rate with ICP-MS during consecutive galvanostatic holds (OER pulses). (f) Total amount of Ir dissolution within the OER pulse. Adapted with permission from ref 2. Copyright 2025 American Chemical Society.

In addition to propane combustion, the hydrogenated Ir_30_pp sample was tested using an electrochemical oxidation reaction (acidic water electrolysis), specifically in the oxygen evolution reaction (OER, Figure 6d). The mass-normalized OER activity increases 8-fold when Ir_30_pp is hydrogenated. Figure 6e,f summarizes the stability of Ir_30_pp against Ir dissolution before and after hydrogenation. It turns out that Ir_30_pp is very stable, even more so than the commercial pure IrO_2_ sample (Ir_100). Upon hydrogenation of Ir_pp_30, Ir dissolution increases by a factor of 3; however, the dissolution rate is identical with that of the commercial Ir_100. Overall, hydrogenation of Ir_30_pp improves the OER activity substantially without compromising the stability.

## 5 Discussion and Outlook

In the typical preparation of supported metal catalysts, the hydrogenation step is performed last to reduce the produced metal oxide particle to the desired metal particle. These studies reveal that the hydrogenation step performed at a specific temperature can be utilized to shape the catalytic oxidation activity. Simple hydrogen treatment can affect mixed oxide catalysis very differently, depending on the applied reduction temperature. For efficient H_2_ dissociation at the surface, one of the components in the mixed oxide must be able to readily dissociate H_2_, which, in our case studies, is either Ru or Ir. The other component in the mixed oxide must stabilize the mixed oxide (LFO or r-TiO_2_) against structural disintegration. This synergistic effect is important for the functionality of the mixed oxides and their overall structural integrity. Ultimately, both types of H_2_ treatment, H incorporation and hydrogen-induced exsolution, lead to *metastable* catalysts that are stable under proper reaction conditions, improving oxidation catalysis of mixed oxides, regardless of whether thermal or electrocatalysis is considered.

The exsolution of Ru from LFRO is a multistep process in which hydrogen acts in various ways. First, hydrogen treatment results in the formation of surface OH due to heterolytic dissociation and its subsequent transfer to the surface O. This combined process is motivated by the study of the hydrogen adsorption on RuO_2_(110).^16^ Further hydrogen treatment leads to water formation at the surface, which can desorb at higher temperature, thus forming an O vacancy on the surface. Oxygen diffusion from the bulk toward the surface leads to reoxidation of the surface and O vacancy penetration to the bulk of LFRO. O vacancies on the surface are continuously produced by the ongoing hydrogen treatment and are filled by oxygen from the bulk. Charge compensation is established by reducing the Ru ion, which increases its size and drives its migration toward the surface. This process is facilitated by the O vacancies. Upon reaching the surface, the Ru ion is fully reduced, forming socketed metallic Ru particles through nucleation. These processes are likely to be of general importance to exsolution and may not be restricted to perovskite oxides. Because these processes must not destabilize the host lattice, perovskite and spinel-like mixed oxides are preferred; however, they are not the only candidates.^29^ The exsolution of LFRO is an ideal subject for first-principles calculations, which can be benchmarked against experimental insights at the molecular level.^44^

The passivating LaO_*x*_ capping layer forms at temperatures above 600 °C, when Ru from the bulk of LFRO begins to exsolve. It has been proposed^3^ that La segregation and precipitation as a LaO_*x*_ covering layer is induced by the overstoichiometry of La in LFRO after Ru exsolution. Upon oxidation at 400 °C, the LaO_*x*_ layer can be reincorporated into the LFRO lattice with a higher binding energy of La than that in the covering layer. Meanwhile, the metallic Ru particles undergo oxidation with a lower surface energy.

For the presented LFRO system, the hydrogen treatment is reversible in that subsequent O_2_ treatment at higher temperatures can restore the original mixed oxides. Therefore, exsolved catalysts are typically employed for high temperature reduction reaction such as those encountered in solid oxide fuel cells at the cathode side^45^ or in ammonia synthesis.^46^ Here, exsolution catalysts have been utilized in oxidation catalysis and in CO_2_ reduction by H_2_ up to temperatures of 500 °C without losing structural stability.^4^ For the CO_2_ reduction the particle distribution greatly impacts the reaction’s selectivity toward CO or methane. For the catalytic propane oxidation reaction at temperatures below 400 °C, the exsolved catalyst is in a *metastable* state because reoxidation of LFRO does not occur.

The size distribution of Ru particles can be adjusted by the temperature during H_2_ exposure, together with a high-temperature redox pretreatment, and therefore by the influx of Ru diffusing toward the surface. We can also purposely decorate the exsolved Ru particles with LaO_*x*_ to avoid further contamination. Only when the catalyst is to be used is the LaO_*x*_ layer removed by briefly annealing the sample to 400 °C in air. The exsolution strategy allows for a high degree of control over the catalyst’s morphology and chemical composition when doping the B sites with various reducible metals.^47^ However, the main obstacle in the exsolution strategy of LFRO is the low surface area of only 10 m^2^/g, which requires sophisticated template-assisted synthesis methods to overcome.

At temperatures between 150 and 250 °C, hydrogen can be dissociated homolytically at the surface to form OH surface groups. Subsequently H from OH can penetrate the bulk of phase-pure mixed oxide of Ru_30_pp and Ir_30_pp, forming incorporated hydrogen. Elevated temperatures are required to overcome the activation barrier for hydrogen penetration into the bulk; for example, the activation barrier for Ru_30 turns out to be about 115 kJ/mol.^1^ The incorporated hydrogen species is labile and can be easily removed by treating with pure O_2_ around 100 °C. However, under reaction conditions where the surface is continuously supplied with hydrogen by dissociation of HCl or propane, the incorporated hydrogen is metastable up to a reaction temperature of 400 °C. Note that the incorporated hydrogen is readily consumed in the CO oxidation process, which serves as a supporting counterexample.^40^ Under electrocatalytic conditions, incorporated hydrogen is stable, as demonstrated by the OER, since the reaction temperature is significantly lower than 100 °C.^2^

The XPS experiments imply that hydrogen penetration into the mixed oxide bulk of Ru_30_pp and Ir_30_pp is not mediated by oxygen vacancies, and the oxidation states of Ti, Ru, Ir, and O are unaffected.^40^ For Ir_30_pp, the formation of oxygen vacancies is directly disproven by electron spin resonance (ESR) experiments.^2^ However, the actual oxidation state of the incorporated H has not yet been determined. For Ru_30_pp a hydride species has been proposed,^1^ while for Ir_30_pp an amphoteric hydrogen species on an interstitial site has been hypothesized based on solid-state ^1^H NMR experiments.^2^ Amphoteric hydrogen can be both protonic (when located closer to O) or hydride species (closer to the metal species) depending on the position in the host lattice.

However, the mechanism by which incorporated hydrogen affects the oxidation activity remains largely unclear. It is likely that the incorporated H alters the electronic structure of the mixed oxide, thereby affecting the catalytic activity either directly or via induced local lattice distortions.^48^ Further first-principles DFT calculations are required to resolve this issue. With time-resolved XRD and MS measurements, the strain evolution and its action on the activity can be followed.^49^ The direct effect of hydrogen incorporation on the electronic structure can be seen in the shift or suppression of the satellite feature in Ru 3d and Ir 4f. To facilitate potential applications, it would be beneficial to stabilize the hydrogen species through co-doping. Knowledge gained from H-storage materials could be beneficial here.^50,51^ The amount of incorporated hydrogen may fine-tune the catalytic performance of mixed oxide catalysts in oxidation and hydrogenation catalysis. Here further studies are required to elucidate this effect and to discover further mixed oxides with a pronounced effect of hydrogen promotion. The class of hydrogen promoted reactions can be expanded to partial oxidation and partial hydrogenation reactions.

Are there links between the two systems I discussed in this Account? At high reduction temperatures (e.g., 800 °C), H_2_ pretreatment is also expected to lead to O vacancy formation. Similar to LFRO, this leads to a phase separation of Ru_30_pp (Ir_30_pp) into TiO_2–*x*_ and Ru (Ir) particles, which are likely covered by a passivating TiO_2–*x*_ layer (SMSI). However, I expect the original r-TiO_2_ structure to collapse. Conversely, low-temperature hydrogen pretreatment at 200 °C has shown to lead to hydrogen incorporation into the LFRO without affecting the oxidation activity in the catalytic propane combustion.^40^ Mild hydrogen post-treatment after exsolution could stabilize hydride ions in the O vacancies formed in LFRO, as has been reported for CeO_2–*x*_.^52,53^ Further research is necessary to understand the formation of incorporated H species in LFRO and their influence on catalytic oxidation and hydrogenation performance.

The hydrogenation of mixed oxides at various temperatures can serve as a general design principle for oxidation catalysis research, whose potential has largely been unexplored. The hydrogenation of mixed oxide catalysts is so straightforward that it should be incorporated into standard screening protocols for catalyst research.
